# The Eastern Pediatric Surgery Network: Creation and implementation of a comprehensive clinical research collaborative in pediatric surgery

**DOI:** 10.1017/cts.2025.10153

**Published:** 2025-09-10

**Authors:** Cornelia L. Griggs, Alyssa Stetson, Aaron M. Lipskar, Mark B. Slidell, Nicole M. Chandler, Christine Finck, William Middlesworth, Shawn J. Rangel, Jason O. Robertson, Hanna Alemayehu, Myron Allukian, Jennifer R. DeFazio, Christina Feng, Matthew A. Hornick, J. Leslie Knod, Afif N. Kulaylat, Sean E. McLean, Jose M. Prince, Mark Puder, Jamie R. Robinson, Robert T. Russell, Stefan Scholz, Anne M. Sescleifer, David J. Hackam, Shaun M. Kunisaki

**Affiliations:** 1 Division of Pediatric Surgery, Massachusetts General Hospital, Boston, MA, USA; 2 Cohen Children’s Medical Center, Division of Pediatric General, Thoracic and Endoscopic Surgery, New Hyde Park, New York, USA; 3 Department of Surgery, Johns Hopkins University School of Medicine, Baltimore, MD, USA; 4 Division of Pediatric Surgery, Johns Hopkins All Children’s Hospital, Saint Petersburg, FL, USA; 5 Department of Pediatric Surgery, Connecticut Children’s Medical Center, Hartford, CT, USA; 6 Division of Pediatric Surgery, Columbia University Irving Medical Center / New York Presbyterian-Morgan Stanley Children’s Hospital, New York, NY, USA; 7 Department of Surgery, Boston Children’s Hospital, Harvard Medical School, Boston, MA, USA; 8 Division of Pediatric Surgery, Cleveland Clinic Children’s Hospital, Cleveland, OH, USA; 9 Division of Pediatric Surgery, Emory University School of Medicine - Children’s Healthcare of Atlanta, Atlanta, GA, USA; 10 Pediatric General, Thoracic and Fetal Surgery, Children’s Hospital of Philadelphia, Philadelphia, PA, USA; 11 Division of Pediatric Surgery, New York-Presbyterian Morgan Stanley Children’s Hospital, New York, NY, USA; 12 Division of Colorectal and Pelvic Reconstruction, Children’s National Hospital, Washington, DC, USA; 13 Division of Pediatric Surgery, Yale New Haven Children’s Hospital, New Haven, CT, USA; 14 Division of Pediatric Surgery, Connecticut Children’s Medical Center, Hartford, CT, USA; 15 Penn State Children’s Hospital, Division of Pediatric Surgery, Hershey, PA, USA; 16 Department of Surgery, University of North Carolina at Chapel Hill, Chapel Hill, NC, USA; 17 Department of Pediatric Surgery, Monroe Carell Jr. Children’s Hospital at Vanderbilt, Nashville, TN, USA; 18 Division of Pediatric Surgery, Children’s of Alabama, Department of Surgery, University of Alabama at Birmingham, Birmingham, AL, USA; 19 Division of Pediatric General and Thoracic Surgery, UPMC Children’s Hospital of Pittsburgh, Pittsburgh, PA, USA

**Keywords:** Consortium studies, pediatric surgery, quality improvement, clinical research, faculty development

## Abstract

Over the past decade, several multi-institutional research consortia have formed within the North American pediatric surgical community. In this article, we describe our experience with the creation and implementation of the Eastern Pediatric Surgery Network, a large and comprehensive research consortium designed to produce a wide array of high-quality clinical studies within our subspecialty. In 2020, a vision statement and rules of governance were established at thirteen academic pediatric surgery divisions in the eastern United States. The research consortium was organized based on four major pillars, namely legal ownership of aggregate data, horizontal leadership structure, mandatory participation in adopted studies, and a broad research portfolio that encompasses the full breath of the specialty. Over the past five years, the number of research projects has dramatically expanded over time and includes participation from 24 different medical centers. Despite a lack of dedicated sponsored extramural support for most projects, there have been 28 abstracts presented at national conferences and 12 manuscripts published in peer-reviewed journals. It is our hope that sharing our experience with creating this organization can help to inform others interested in establishing the academic infrastructure to engage in multi-institutional, evidence-based clinical research in other medical specialties and beyond.

## Introduction

Pediatric surgery is a small yet diverse surgical specialty that treats a broad range of common and uncommon diseases. Historically, a substantial proportion of the pediatric surgical literature has been comprised of single center studies that were retrospective and lacking adequate controls [1]. While these studies can have tremendous value, their results cannot always be widely generalized, and they often failed to produce measurable changes in quality improvement and clinical practice on a broad scale.

In response to these challenges, several multi-institutional research consortia have formed in recent years within the North American pediatric surgical community. The Pediatric Surgery Research Collaborative (PedSRC) and the Midwest Pediatric Surgical Consortium (MWPSC) were both founded in 2013 to facilitate regional and national collaboration [2–5]. The Western Pediatric Surgery Research Consortium (WPSRC) and the Canadian Consortium for Research in Pediatric Surgery were subsequently established in 2018 with similar missions [6–8]. We have also witnessed the proliferation of numerous disease-specific pediatric surgery research groups including the Congenital Diaphragmatic Hernia Study Group, the ATOMAC+ Pediatric Trauma Research Network, the Pediatric Colorectal and Pelvic Learning Consortium, and the Pediatric Surgical Oncology Research Collaborative [9–12]. Collectively, these organizations have served as important vehicles for advancing the quality and translatability of clinical pediatric surgical studies through their practice-changing work on topics such as managing tracheoesophageal fistulas, predicting choledocholithiasis, and treatment for high-risk Ewing sarcoma [13–15].

In this paper, we describe our experience creating and implementing the Eastern Pediatric Surgery Network (EPSN), the largest regional PedSRC within the current academic landscape. We also aim to highlight the operational aspects of EPSN that we believe distinguish this consortium and have made it effective for conducting innovative collaborative studies to advance pediatric surgical care.

## Methods

Inspired by the early success of the MWPSC [3,16,17]. EPSN was conceived in June 2019 to increase clinical research collaboration among pediatric surgeons located in the eastern United States. A SWOT (strengths, weaknesses, opportunities, and threats) business model analysis was adopted before developing a vision and a set of core values for the new organization. Strengths included the high density of established academic pediatric surgeons and hospitals in the eastern United States, with recognized expertise in either specific disease management or research methodology. An additional and notable strength was the heterogenous array of institutions in the region, including northern and southern states, smaller and larger pediatric hospitals, urban and rural populations, and centers with and without pediatric surgery training programs. Weaknesses of this new consortium were potential for distrust and competition among institutions, often fueled by close geographical proximity. Recognized opportunities were the chance to unite a greater number of hospitals when compared to other collaboratives, the ability to drive practice change for many unsolved universal and rare clinical problems, the possibility of gaining relevance and becoming a major stakeholder at national meetings, and the potential to successfully obtain external grants to support clinical research in the current hyper-competitive funding environment. Looming threats were a lack of sustainability due to decentralized resources and potential for duplicative efforts, especially if studies overlap with ongoing projects at member institutions or those in other consortia.

In August 2019, division chiefs from some of the largest academic children’s hospitals on the East Coast convened through a series of teleconference meetings where the rationale, mission, vision, and process for the establishment of a new broad-based clinical research consortium were discussed. Attendees also included members from a statewide regional quality improvement consortium and the Johns Hopkins central institutional review board (IRB) panel. Multiple topics related to the rules of governance were discussed and, when majority opinion was reached, were organized along four major pillars: legal ownership of aggregate data, horizontal leadership structure, mandatory participation in adopted studies, and a broad research portfolio that encompasses the full breath of the specialty (Table 1). A consortium agreement charter was drafted in November 2019 and the acronym for the organization (EPSN) was coined.

**Table 1.** Rules of governance, January 2020

Legal
Common document initiated by Johns Hopkins School of Medicine - Signed by institutional officials from all member sites - Requires renewal every five years
Each member site owns its institutional data but not the aggregate data
Secure, cloud-based data management (REDCap) system with limited data set preferred
Central IRB process encouraged
No consortium dues

Leadership
One designated surgeon representative (board member) per site
Horizontal structure with each board member having equal representation
Officers (2-year term) elected by the board by simple majority
Chair and Vice Chair cannot be from same institution

Participation
Commitment to participate in all studies with exceptions allowed on a case-by-case basis
Group conferences every two months with mandatory attendance from board members
At least one in-person meeting per year, rotating among member sites
Lead institution agrees to form a working group with those outside of institution
At least two authors per site allowed under ICMJE guidelines
Any faculty from any member institution can submit a proposal and serve as lead investigator
Site of lead investigator serves as the central data coordinating center

Content
No limitations on disease process or type of research
Adoption of new projects after blinded review and majority vote

One representative from each institution was designated to serve as an accountable board member. A list of EPSN member institutions is shown in Table 2, with the founding centers designated. Institutional member representatives (board members) comprised a diverse group regarding gender (31% female), race/ethnicity (15% underrepresented) [18], and research interests.

**Table 2.** Member hospitals of the Eastern Pediatric Surgery Network, December 2024

Participating Centers	Pediatric Surgery Fellowship	Estimated Bed Capacity
Boston Children’s Hospital/Harvard (Boston, MA)*	Yes	485
Children’s Healthcare of Atlanta/Emory (Atlanta, GA)*	Yes	446
Children’s Hospital of Philadelphia/Penn (Philadelphia, PA)*	Yes	695
Children’s National Medical Center (Washington, DC)	Yes	323
Children’s of Alabama (Birmingham, AL)	Yes	332
Cleveland Clinic Children’s Hospital (Cleveland, OH)	No	236
Cohen Children’s Medical Center (Manhasset, NY)*	Yes	187
Connecticut Children’s Hospital/UConn (Hartford, CT)*	Yes	203
Duke Children’s Hospital & Health Center (Durham, NC)*	No	202
Hassenfeld Children’s Hospital at NYU Langone (New York, NY)	No	102
Johns Hopkins All Children’s Hospital (St. Petersburg, FL)*	Yes	259
Johns Hopkins Children’s Center (Baltimore, MD)*	Yes	204
MassGeneral Hospital for Children/Harvard (Boston, MA)*	No	100
Monroe Carell Jr. Children’s Hospital at Vanderbilt (Nashville, TN)	Yes	363
Morgan Stanley Children’s Hospital/Columbia (New York, NY)*	Yes	269
Penn State Children’s Hospital (Hershey, PA)	No	134
UF Health Shands Children’s Hospital (Gainesville, FL)	Yes	208
University of North Carolina Children’s Hospital (Chapel Hill, NC)*	No	150
University of Rochester-Golisano Children’s Hospital (Rochester, NY)	No	132
University of Virginia Children’s Hospital (Charlottesville, VA)	No	112
UPMC Children’s Hospital of Pittsburgh (Pittsburgh, PA)*	Yes	327
Yale New Haven Children’s Hospital (New Haven, CT)*	Yes	202

* Denotes founding member.

## Results

### Consortium growth

**Figure 1.**
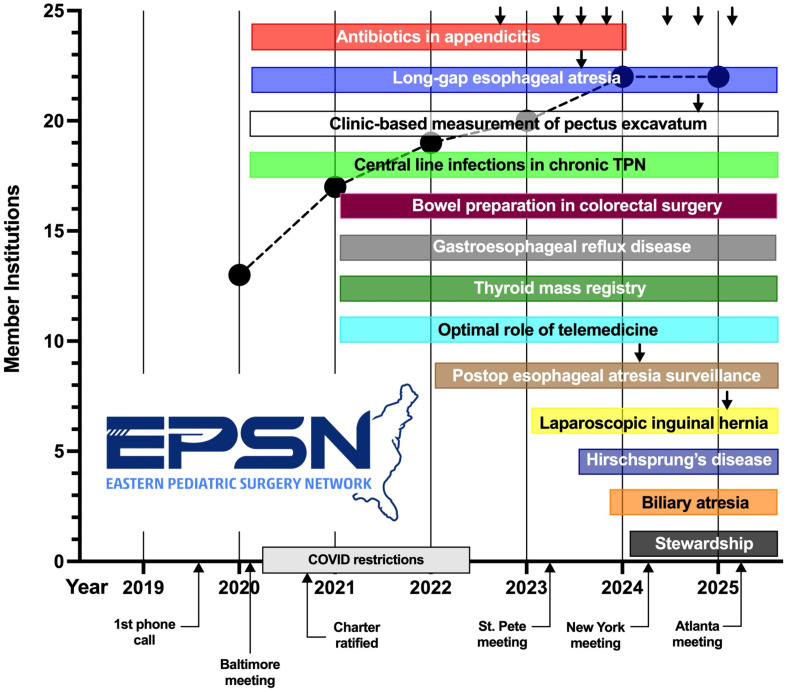
Eastern Pediatric Surgery Network (EPSN) timeline of consortium projects (listed in boxes) and membership institutions (black circles, Y-axis) as of December 2024. Black arrows indicate published manuscripts.

At the inaugural meeting in January 2020 in Baltimore, Maryland, 24 study proposals were submitted, and four (17%) were ultimately selected for adoption by EPSN after blinded review and vigorous debate. Six weeks later, restrictions imposed due to the COVID-19 (SARS-CoV-2) pandemic resulted in a stoppage of most or all research activities, including IRB review, across all member institutions. Despite pandemic-related challenges, progress on research projects regained momentum with group meetings converted entirely to a video-based platform. A new informational website (www.easternpediatricsurgery.org) was created in August 2020, and the consortium charter was ratified by the institutional representatives of all founding centers in September 2020. An organizational logo was created by a third-party graphic designer in December 2021 (Figure 1).

Given our vision to conduct large multi-institutional studies for a wide range of conditions managed by pediatric surgeons, we admitted between one and four additional institutions annually, provided that they could provide evidence of an adequate research infrastructure and a demonstrated track record of scholarly activity. This approach has led to membership expansion from 13 to 22 hospitals as of December 2024 (Figure 1). The mean bed capacity of member hospitals is now 260 (range 102–695). While all centers are university-based and have well-established general surgery residency programs, seven (32%) do not have an accredited pediatric surgery fellowship training program. The mean number of pediatric surgeons per center is 9.2 (range 3–22) (Table 2).

The efficiency and ease afforded by video conferencing meant that that virtual meetings could be held bi-monthly and that in-person meetings should then be conducted on an annual basis once COVID restrictions were lifted. This meeting schedule was designed to balance consortium identity and momentum with the time burden that accompanies more frequent in-person encounters. The second annual meeting was held in February 2023 at Johns Hopkins All Children’s Hospital in St Petersburg, Florida, and the third was held in February 2024 at Cohen Children’s Medical Center/Northwell Health in New York.

Since the consortium’s inception, there has been a steady increase in the number of participating surgeon investigators across the different projects. The inaugural in-person meeting included 13 pediatric surgeons and one trainee in attendance from ten founding institutions. At the most recent annual meeting held in February 2025 at Children’s Healthcare of Atlanta (CHOA), there were 21 pediatric surgeons and 13 trainees who attended in person from 18 member institutions. With mounting engagement and continued growth of the consortium, we have had the opportunity to engage and mentor junior faculty (e.g., assistant professors, those less than five years from fellowship completion), who bring innovative ideas to the group, and have subsequently become lead principal investigators on consortium projects. Substantial medical student and resident involvement from institutions leading individual studies is demonstrated by their inclusion as first authors on many presentations and manuscripts to date.

### Consortium studies

Based on our core principles, high standards and accountability have been paramount, and the portfolio of EPSN studies reflects a wide spectrum of pediatric surgical disease. Two of the initial projects adopted were retrospective in nature: (1) postoperative antibiotic use in complicated appendicitis and (2) management of long-gap esophageal atresia (EA). The appendicitis study has since been completed, and the long gap study is moving from a retrospective to a prospective phase. The other initial projects that were adopted, (3) validating clinic-based measurements of severity in pectus excavatum and (4) streamlining practices to rule out central line-associated bloodstream infection in patients on chronic total parenteral nutrition, both prospective studies, have recently closed to further enrollment. The retrospective cohort studies had fewer IRB approval obstacles, making them more likely to be presented and published within a short timeframe, whereas the prospective studies allow us to set more ambitious, longer-term project goals that involve robust databases. Moreover, we favored initial projects where there was an existing research infrastructure for the project at the lead institution and a preliminary manual of operations with pilot data to demonstrate study feasibility [19–21]. Due to the inherent heterogeneity in the data elements collected for a given project as well as the high administrative costs associated with a single IRB process at most institutions, we elected to handle all but one at the local IRB level.

Between one and four new research projects have been adopted in each subsequent year, resulting in 13 ongoing or completed projects by the end of 2024 (Figure 1). A wide variety of topics, indicative of the diversity of clinical pediatric surgery, are now being studied. Current projects focus on thyroid nodules, surgical management of gastroesophageal reflux disease, biliary atresia, laparoscopic repair of inguinal hernia, bowel preparation in colorectal surgery, and perioperative antimicrobial stewardship, among others. The disruption in standard workflow caused by the COVID lockdowns inspired a multi-dimensional study on the optimal role of telemedicine in the post-pandemic practice environment. We have found that the tempo of adding a small number of vetted proposals on an annual basis prevents overburdening member institutions yet still encourages new ideas and the introduction of diverse and impactful research. We have favored projects where there might be an opportunity to answer multiple different research questions once a multi-institutional database has been established.

The innovative culture fostered by our framework has allowed for the introduction of several approaches to consortium-based research that, to our knowledge, are not well described. One specific example has been the creation of novel datasets by supplementing quality improvement databases, such as the American College of Surgeons National Surgical Quality Improvement Program Pediatric (NSQIP-P), with more granular data extracted from electronic medical records at the institutional level [22]. This methodology, extensively used in our appendicitis project, substantially expedited an otherwise laborious data collection process. Another approach has been the endorsement of survey-based studies to inform and design subsequent retrospective or prospective cohort studies. Not only does this provide us with baseline data for planned cohort studies, but the surveys performed thus far have demonstrated high response rates and have been published as stand-alone manuscripts in peer-reviewed journals [23,24].

### Research productivity

Despite the challenges associated with COVID restrictions on our fledgling organization, overall research output has steadily increased based on several metrics. The first consortium abstract, which focused on operative reports and disease severity in appendicitis, was presented less than two years from study inception (Figure 1). A total of twelve studies have been published in peer reviewed journals at the time of this manuscript preparation [22–32] (Table 3). Although pediatric surgery subspecialty journals have appropriately been a common destination for our work, over half of EPSN manuscripts have been published in surgical journals that have a readership beyond that of the pediatric specialty. Twenty-three abstracts have been presented at national conferences, including three plenary talks at the 2025 American Pediatric Surgical Association Annual Meeting. As expected, patient/provider surveys and retrospective studies based on administrative databases have been easier to complete in a short time span when compared with prospective studies. A handful of our adopted projects have proven more challenging due to administrative/staffing reasons or reliance on non-surgical collaborators within each institution. As our research base continues to broaden, we have begun to study biliary atresia, the rarest of pediatric surgical diseases, in collaboration with MWPSC and WPSRC. Thus far, our consortium has been primarily funded by philanthropic support and internal departmental funds within each division. We also have relied on research coordinators at each lead site to manage the individual projects.

**Table 3.** Manuscripts published from EPSN, December 2024

First Author	Date	Title	Journal
Fell	2020	Optimizing Duration of Empiric Management of Suspected Central Line-Associated Bloodstream Infections in Pediatric Patients with Intestinal Failure.	Journal of Pediatrics
Cramm	2022	Association of Gangrenous, Suppurative, and Exudative Findings with Outcomes and Resource Utilization in Children with Nonperforated Appendicitis	JAMA Surgery
Cramm	2022	Predictive Value of Routine WBC Count Obtained Before Discharge for Organ Space Infection in Children with Complicated Appendicitis: Results from the Eastern Pediatric Surgery Network	American College of Surgeons
Cramm	2023	Association Between Antibiotic Redosing Before Incision and Risk of Incisional Site Infection in Children with Appendicitis	Annals of Surgery
Finck	2023	Perioperative management and outcomes in long-gap esophageal atresia: A retrospective analysis from the Eastern Pediatric Surgery Network	Journal of Pediatric Surgery Open
Hamilton-Hall	2023	Esophageal Surveillance Practices in Esophageal Atresia Patients: A Survey by the Eastern Pediatric Surgery Network	Journal of Pediatric Surgery
Cramm	2023	Outcomes and Resource Utilization Associated with Use of Routine Pre-Discharge White Blood Cell Count for Clinical Decision-Making in Children with Complicated Appendicitis: A Multicenter Hospital-Level Analysis	Journal of Pediatric Surgery
Cramm	2023	Use of Antipseudomonal Antibiotics is Not Associated with Lower Rates of Postoperative Drainage Procedures or More Favorable Culture Profiles in Children with Complicated Appendicitis	Annals of Surgery
Cramm	2024	Utility of a Benchmarking Report for Balancing Infection Prevention and Antimicrobial Stewardship in Children with Complicated Appendicitis	Annals of Surgery
Cramm	2024	Postoperative Antibiotics, Outcomes, and Resource Use in Children with Gangrenous Appendicitis	JAMA Surgery
Green	2024	Measuring Pectus Excavatum Severity, External Caliper, or Cross-Sectional Imaging: Family Perceptions	Journal of Surgical Research
Hellmann	2024	Preferences for Inguinal Hernia Repair in Infants: A Survey of the Eastern Pediatric Surgery Network	Journal of Surgical Research

## Discussion

Over the last decade, clinical research consortia seeking to enrich the quality of outcomes research have proliferated in the specialty of pediatric surgery [33]. From a clinical perspective, these studies build on established work with quality improvement databases and disease-specific registries to yield sizable cohorts of patients with adequate statistical power and more generalizable results to help inform clinical practice [34]. This is particularly important for rare congenital anomalies, which are the specialty-defining disorders treated by pediatric surgeons [3,17,35]. Historically, surgeons have relied on single center studies, often from our national quaternary care centers, or disease-specific collaboratives assembled by centers of excellence or expert thought leaders in the field. Although such studies can be invaluable, some findings may have limited generalizability to pediatric surgeons who are less specialized, or who practice in smaller clinical settings [36]. While administrative and quality improvement databases provide another way to capture multi-institutional data, they are subject to coding errors, may not have essential data to adjust for confounders, and are vulnerable to selection bias since they may not be specifically designed to answer the proposed clinical research question. In this paper, we describe the framework and our early experience building a cohesive and successful East Coast regional clinical research consortium designed to address a broad range of clinical questions in pediatric surgery.

While the development of pediatric surgeon-based clinical research collaboratives aimed at robust, high-quality multi-institutional studies is certainly not new [2,8,11], we argue that there remains a compelling need for broad-based research organizations that extend beyond a limited region to complement existing regional and disease-specific networks. There are also several specific aspects of our consortium establishment that are worthy of mention. First, the timing of our establishment in early 2020 meant that our organization was largely conceived during restrictions imposed by the COVID pandemic (March 2020 to May 2023). The “silver lining” of the pandemic was the rapid acceptance of the virtual meeting platform, which paradoxically allowed us to gain more familiarity with each other through increased attendance, thereby fostering a culture of camaraderie, transparency, and respect. The assimilation of new centers into the organization by video conferencing was straightforward, and virtual meetings can lower barriers to entry for trainees and smaller institutions, with more limited academic resources, to otherwise fully engage in the consortium process [37]. It is difficult to imagine how effective communication, data sharing, and growth of such a large group of surgeons could have occurred in the pre-2020 era of teleconferencing. Video conferencing has also provided us more flexible scheduling at a lower cost than in-person encounters. Although face-to-face interactions still represent the gold standard for academic collaborations in surgery, technological advances and shifting cultural norms in modes of communication within the past few years have clearly led to permanent changes in workflow for major scientific organizations. Moreover, a nontrivial percentage of our pediatric surgeons have found it difficult to meet in person, given the wide array of professional conferences already offered throughout the year on top of clinical, family, and personal obligations.

Within our horizontal leadership structure based on inclusivity, we have developed frameworks early on in our foundation that openly welcome other surgeons within member hospitals in an “a la carte” fashion for specific studies. Many of these individuals have nationally and internationally recognized expertise from a methodological or disease process perspective (e.g., quality improvement, implementation science, complex esophageal surgery) but do not have the bandwidth or the desire to engage in a wide array of clinical studies. Nevertheless, these content experts have been co-authors commensurate with their contributions to specific studies.

Through the establishment of our consortium, we have witnessed a surprising willingness of members to overcome silos that have historically existed among academic pediatric institutions, particularly in the Northeast and Middle Atlantic regions of the country. Such silos were perpetuated in some instances by distrust and/or close geographic proximity with resultant competition for the same high-density patient base. Some EPSN institutions are located within the same metropolitan area yet had never collaborated on research projects before in a meaningful way. It was particularly critical as a collaborative to gain the trust and solidarity of chairs from the larger pediatric surgical programs, since they might have strong incentives to publish single institutional studies that promote a dominant hospital brand in the healthcare marketplace.

Existing barriers to fruitful collaboration have been overcome using a multipronged approach. At larger institutions, we observed an increasing awareness of the limitations of single center studies to drive practice change at other institutions. For medium-size and smaller academic divisions, there is a desire to remain involved in high-quality studies, given the known limitations of administrative and quality improvement data sets as well as the increasing sophistication of statistical methods employed. Since the data-coordinating center is based at the lead institution for each project, lead institution autonomy is largely preserved. We also speculate that many division chiefs have embraced the value of a shared clinical research space where experienced surgical investigators can closely mentor enthusiastic junior faculty and trainees with mutual research interests, regardless of institution. It is the hope that the benefits of EPSN participation will help these younger surgeons along their path to leadership in academic pediatric surgery.

For multiple reasons beyond the scope of this paper, there has been a major shift in academic focus among pediatric surgeons away from basic science endeavors and towards clinical outcomes research [38]. Clinical research consortia with a broad interest in various pediatric surgical problems therefore provide a critical avenue for many less specialized surgeons and trainees to academic advancement [39]. The large number of authors required to perform many consortium-based studies is openly acknowledged at project inception, and more importantly, is allowed by many journals so long as there is adherence to authorship guidelines set forth by the International Committee of Medical Journal Editors (ICMJE). Written guidelines of our group authorship model, including the role of nonauthor collaborators, are delineated in the Appendix section of the signed member agreement. Finally, academic surgeons have realized that an established research infrastructure allows meaningful participation in regional clinical research, which may in turn provide opportunities to be involved in national clinical studies. For our rarest surgical conditions, the formation of nationwide studies to accrue meaningful numbers of study subjects seems imminent.

To ensure that study conclusions are generalizable we have prioritized diversity in the size and character of participating centers. This has led to a patient referral catchment area encompassing over 17 states and the District of Columbia, ranging from Florida to Massachusetts and as far west as Alabama and Tennessee. Sites are located in urban, suburban, and rural areas, thereby representing a heterogeneous patient population. Our consortium contains two of the largest freestanding children’s hospitals in the country alongside many smaller divisions that are well-integrated components of general health systems. Geographic location within the country and participation in other research consortia have not been disqualifiers for application to EPSN. Our strategy of measured institutional expansion over time does have its own limitations (as larger groups may be unwieldy to organize and manage) but may allow greater external validity when translating research findings across a broader range of pediatric surgical care settings.

Our consortium stands at a critical inflection point in terms of the opportunity to gain momentum and increase productivity in the years to come. Numerous challenges need to be addressed to ensure the long-term viability of the organization. First, securing multi-million-dollar external grants represents an important next step towards sustainability and would be a prerequisite to organizing large scale prospective clinical trials, implementation studies, and innovative basic science research using patient samples from biorepositories. Second, following the lead of the other regional research consortia, we have recently become a non-profit business entity (501c3), which will enable us to collect institutional membership dues and donations to fund shared research activities. Third, we need a central research coordinator, funded by the consortium, who will effectively execute multidisciplinary projects across the large number of institutions involved. This person would also serve as a centralized resource inherent to operationalize more complex IRB and data use agreements and ensure equitable resource allocation and optimized research capacity across participating divisions.

Continued investment in multi-institutional collaboratives will likely make a substantial impact in the evidence-based practice of pediatric surgery. However, there needs to be national conversation among pediatric surgeon leaders of the various regional consortia and disease-specific collaboratives aimed at reducing undesirable competition, improving efficiency of studies, avoiding duplicative research efforts, and advancing the adoption of evidence-based practices in our field [40]. Such an approach, which has already been established among pediatric surgeons in Canada [8], seems necessary for solving common administrative challenges intrinsic to multi-institutional research collaboratives and for generating the statistical power required for some of the least common yet most complex pediatric surgical conditions that have high mortality and/or morbidity. Future directions include a business plan for sustainable growth and development, as well as a call for greater national collaboration to better understand some of the most pressing clinical problems that plague the patients and families that we serve.
